# Teachers’ Perceptions of Student Mental Health in Eastern China: A Qualitative Study

**DOI:** 10.3390/ijerph18147271

**Published:** 2021-07-07

**Authors:** Min Yao, Paul I Kadetz, Aissata Mahamadou Sidibe, Yedong Wu, Jiameng Li, Jinping Lyu, Cuiling Ma, Therese Hesketh

**Affiliations:** 1 Center for Global Health, Zhejiang University, Hangzhou 310058, China; 21818501@zju.edu.cn (M.Y.); paulkadetz@gmail.com (P.I.K.); a.sidibe@zju.edu.cn (A.M.S.); wuyedong@zju.edu.cn (Y.W.); jiamengli@zju.edu.cn (J.L.); 21818502@zju.edu.cn (J.L.); 21918697@zju.edu.cn (C.M.); 2 Center for Executive Education, University of Global Health Equity, Kigali P.O. Box 6955, Rwanda; 3 Institute for Global Health, University College London, London WC1N1EH, UK

**Keywords:** China, adolescents, mental health, behavior, medicalization, stigmatization, *ban zhu ren*

## Abstract

In China, primary and secondary school teachers, known as *ban zhu ren*, have pastoral responsibility for the students in their class. The aim of this preliminary study is to identify how *ban zhu ren* perceive the mental health of their students, and how they have acted on these perceptions. Content analysis was used to organize the data and distinguish categories or themes derived from in-depth semi-structured interviews conducted with 27 *ban zhu ren* from Zhejiang and Anhui provinces. Frequencies of informant responses were used to identify the areas of agreement and disagreement across identified categories and themes among the informants. The results illustrate that the informants consider issues, such as not paying attention in class (*n* = 14), not getting along well with classmates (*n* = 12), and excessive gaming (*n* = 11) to be indicative of mental illness, although these would commonly be considered normal adolescent behaviors. Fifteen informants admitted that they found it difficult to work with student mental health issues, and 18 felt they had inadequate or non-existent training. However, all informants stated that they had intervened with what they perceived to be students’ mental health issues, although only 9 informants had referred students for professional help. The informants reported that they were reluctant to provide referrals, due to the stigmatization they believed students would experience if given a diagnosis of mental illness. We conclude that among our informants there is a lack of agreement on what behavioral and mental health issues are, and that informants may be confusing what are, in actuality, non-conformist or non-compliant (yet often normal), adolescent behaviors with mental illness due to insufficient mental health training.

## 1. Introduction

In 2019, there were 1.2 billion adolescents, aged 10–19 years, representing 16% of the world’s population [1]. It is estimated that approximately 20% of the world’s adolescents experience mental health issues [2]. According to recent WHO data, the most common mental health problems for adolescents include emotional disorders, such as depression and anxiety, eating disorders, psychosis, and self-harm [3]. Mental health problems are the leading cause of disease burden among young people [4]. Suicide was the third most common cause of adolescent mortality in 2015, resulting in an estimated 67,000 deaths worldwide [5]. Mental illness in adolescents is reported to be increasing in many countries [6]. This is of particular concern because mental health issues during adolescence are known to be a predictor of long-term mental well-being [7].

In China, there are growing concerns about increasing mental health problems in adolescents. A recent nationwide study among Chinese adolescents aged 12 to 18 years estimated that the prevalence of mild, moderate, or severe symptoms of anxiety and depression was 43.7% and 37.4%, respectively [8], with estimates for self-harm of 27.6% and attempted suicide of 7.3% [9,10]. Hence, diagnosis and appropriate management of mental illness in Chinese adolescents is of the utmost importance. Since the overwhelming majority of Chinese adolescents attend school, schools are an appropriate environment in which to identify mental health issues and to provide support when necessary. The responsibility for this support may therefore fall on teachers.

*Ban zhu ren* are primary and secondary school teachers who are responsible for all aspects of student education in China. Their role is closest to what are called “class teachers”, “homeroom teachers” or “classroom tutors” in other countries, although they teach in areas that range from mathematics to English and Chinese [11]. *Ban zhu ren* are put through a selective process that seeks “qualified” teachers who can take on additional responsibilities [11]. They are generally selected for their personal suitability for the role and will receive specific training. The training includes: (1) professional theory, legal standards for teachers, compulsory education law, review of both job responsibilities and the school’s work guide; (2) professional literacy, including organization and leadership; and (3) building professional abilities, including collective organization and management, emergency response, and the observation, education, and analysis of student abilities. Before a teacher can become a *ban zhu ren*, they must also receive initial training that aims to improve their ability to manage the classroom.

According to the Ministry of Education’s 2009 report, “Regarding the Responsibilities of Ban zhu ren in Primary and Middle Schools”, *ban zhu ren* are expected to: (i) have a comprehensive understanding of each student in their class, including their capacity to learn, their daily life, and their mental well-being, in order to help students reach their “full potential”; (ii) manage the class and create a good study environment for students; (iii) organize class activities, such as class meetings, field trips, and other off-campus activities; and (iv) communicate frequently with other teachers and parents about a student’s progress [12]. In general, “*ban zhu ren* are supposed to be the leader, the organizer, and the educator of a class, who are in charge of facilitating students’ academic study, implementing classroom discipline, organizing extracurricular activities, and maintaining contact with subject teachers and parents involved in their class” [13].

In 2012, the Chinese Ministry of Education declared a need for psychological services in primary and secondary schools, due to a reported worsening of student mental health [14]. As a result, every primary and secondary school in China is required to have at least one full-time or part-time psychological counselor who is responsible for the psychological well-being of students [14]. Furthermore, mental health education is to be included in the school curriculum. However, there is no systematic training before becoming a psychological counselor in Chinese schools [15]. Therefore, in many schools, the *ban zhu ren* are expected to take responsibility for mental health education, by offering classes that improve mental health awareness, identifying mental health problems in students [16,17], and referring students for professional mental health services when necessary [18,19,20]. 

China is not unusual in this regard. Schools have been identified globally as an important environment for the support of adolescent mental health [21,22,23]. For example, an Australian study details the duty that teachers felt they had to identify and refer students who might be experiencing mental health issues [24]. Similarly, in the US, teachers are responsible for identifying and providing appropriate support to students with mental health issues [25,26]. Thus, a teacher’s responsibilities for student mental health are not unique to *ban zhu ren* in China.

Much of the literature reviewed, broadly identifies how teachers in China can impact student mental health. For example, according to Luo et al., (2011), ”The positively nurturing interpersonal relationships between teachers and students play an important role in student success and found that emotionally warm relationships between teachers and students provide students with a sense of security within school” [27]. Other examples of teacher support benefiting student mental health [28,29,30] include Wang et al., who conclude that creating a classroom environment conducive to improve student mental health should be a goal for all teachers [31]. 

The specific role of teachers as providers of student mental health support varies across countries and settings [32,33]. For example, where, in China, the *ban zhu ren* have the responsibility to refer students to mental health professionals [34], in some settings, such as in Turkey, there is a greater reliance on school psychologists for professional referral [35]. Several studies have demonstrated that teachers in Chinese schools feel they lack the experience and training to manage students’ mental health problems and require more practical training and mentorship in mental health [36,37]. Ren et al. also identified a need for cooperation between teachers and psychologists in Chinese schools in order to identify student mental health risks in a timely manner [38]. In general, adolescents in Chinese schools are thought to benefit from mental health education, and schools are recognized as suitable venues for mental health education and mental health services for adolescents [39,40]. Thus, the school environment, and the relationship between teachers and students, are important determinants of student mental health. 

Even though the *ban zhu ren* assume an important role in supporting student mental health, few, if any, studies examine the perceptions, knowledge, and/or activities of *ban zhu ren* concerning student mental health. The aim of this research is to identify how a group of *ban zhu ren* perceive the mental health of their students, and how they have acted on these perceptions. Specifically, this study seeks to identify patterns of meaning across the data, including: (i) how *ban zhu ren* differentiate between behavioral and mental health issues; (ii) what *ban zhu ren* do when they believe a student has mental health issues; and (iii) the perception *ban zhu ren* have of their ability to effectively manage these issues. 

## 2. Research Methods

### 2.1. Study Design

This qualitative study employed in-depth semi-structured interviews with a convenience sample of 27 *ban zhu ren*, and content analysis was employed to organize and interpret the data.

### 2.2. Site and Informant Selection

This study was conducted with *ban zhu ren* from middle schools (ages 12 to 15) in Zhejiang and Anhui provinces, which represent relatively wealthier and poorer provinces, respectively. Anhui is an inland eastern province with a population of 63.7 million and is ranked at 13 out of 31 provinces for GDP per capita, whereas Zhejiang is an eastern coastal province with a population of 58.5 million and ranked fifth in the nation for GDP per capita [41,42]. Using a convenience sampling process (ie; schools were chosen according to the researchers’ affiliation with teachers at the schools, who acted as intermediaries between the researchers and school administration), a total of 6 middle schools were selected from Anhui province with: 2 middle schools from the provincial capital, 2 from the city level, 1 from the county level, and 1 from the township level. In Zhejiang province, only 1 middle school from the provincial capital, 1 from the city level, and 1 from the township level were included in this research, due to COVID-19 pandemic restrictions. Hence, a total of 9 middle schools were included in this research.

A convenience sample of 1 *ban zhu ren* from each grade cohort, or 3 informants from each of the 9 schools, were recruited. Inclusion criteria were being the *ban zhu ren* of the class and having more than one year of work experience. In total, 27 *ban zhu ren* from 9 middle schools were eligible for inclusion. In one school in Zhejiang province, 2 *ban zhu ren* from the eighth-grade class, and 1 *ban zhu ren* from the seventh-grade class were included, because the *ban zhu ren* from the ninth-grade class were not available. *Ban zhu ren* were recruited with the help of the teacher intermediaries, who introduced the researcher (YM) to the *ban zhu ren* at their school. *Ban zhu ren* were then invited to participate in the research and those who agreed were included in the study.

### 2.3. Data Collection

A topic guide based on a review of the literature served to generate the original interview schedule, containing a broad range of questions, for an initial pilot study of semi-structured interviews conducted with 6 *ban zhu ren*. Following an iterative review of the initial interview schedule with members of the team (T.H., P.I.K., M.Y.), modifications were then made to the final interview guide, with several questions combined and with two questions about interventions for student mental health added, and one question, that was unclear to initial pilot informants, was removed.

The final interview schedule had 24 open-ended questions (see Table S1). In-depth semi-structured interviews were conducted from April to May 2020. Interviews lasted between 40 and 90 min and were conducted by a trained researcher (M.Y.). The interviews were conducted in Chinese. Translation, between a native Chinese speaker (M.Y.), a researcher proficient in English and Chinese (A.M.S.), and a native English speaker (P.I.K.), followed a lengthy iterative process to generate the most accurate interpretation of the data. Specifically, all data was translated and then coded by YM, first, prior to English translation by A.M.S. and P.I.K. 

Before each interview, informants were asked to complete a questionnaire on demographic information including their province, sex, age, type of school, the grade they taught, years teaching, years as a *ban zhu ren*, the number of students in their class, and subject taught (see File S1). After introducing the study to each informant and obtaining verbal informed consent, the interview was conducted either via WeChat (Tencent, Shenzhen, China; a popular Chinese social media platform) or phone call, according to the informant’s preferences, due to COVID-19 pandemic restrictions.

The interviews primarily focused on: (i) identification of mental health issues in students; (ii) differentiation of behavioral issues from mental health issues; (iii) support of students with mental health issues; and (iv) perceived ability to provide appropriate support. All interviews were recorded and transcribed prior to data analysis.

### 2.4. Data Analysis 

The data collected for this research were the informants’ responses to open-ended questions in semi-structured interviews. In order to organize informant responses in a way that provided meaning, content analysis was employed to analyze the data. Since we were interested in identifying and grouping responses according to content that shares a commonality, and according to their frequencies among informants, both conventional and summative content analyses were used. Summative content analysis facilitates both the creation of categories and the quantification of qualitative data [43,44]. Conventional content analysis was also appropriate here, as it is used for research that is novel and not built on existing research, in which case an inductive process of content analysis is employed. “An approach based on inductive data moves from the specific to the general, so that particular instances are observed and then combined into a larger whole or general statement”, as illustrated in Table 1 (which should be read from the right column to the left column) [43]. 

Content analysis is a nonlinear, iterative process of analysis. In this analysis, data were grouped using an iterative and (predominantly) inductive process, closely linked to the semantic data content. Specifically, the data-based meaning was attributed using an inductive approach, while theory-based meaning (such as medicalization) employed a deductive approach.

The researchers conducting data analysis (P.I.K., M.Y. and A.M.S.) pursued the following steps in this summative and conventional content analysis [43,44]. (1) Researchers were familiarized with the data by repeated readings of interview transcripts and initial highlighting of sections of relevance, to answer research questions. (2) Researchers identified key words, thoughts, or concepts in the text that appeared with frequency and that provided an “understanding [of] the contextual use of the words or content” [44]. (3) Researchers generated initial descriptive and interpretive codes from the key words, thoughts, and concepts, which were grouped according to each research question. (4) Codes were then grouped into categories or themes, based on the relationship of different codes to the research questions. During this step, researchers engaged in an iterative process of reviewing the accuracy and effectiveness of categories/themes; first in relation to the codes, and then in relation to the entire data set. During this process, several of the original categories that were redundant or significantly overlapped were combined to form the final seven categories/themes. The categories/themes were logically ordered to “tell the story” of the research. (5) Categories/themes were organized into four group clusters or content areas of the informant’s perception of, differentiation of, interventions in, and capacity for handling, student mental health issues.

All aspects of data analysis were conducted without software and after agreement regarding the interpretation of the data by the contributing authors. A summary of the final categories and sub-categories is detailed in Table 1.

### 2.5. Ethics

Ethical approval was obtained from the Ethics Review Committee of Zhejiang University (ZGL201909-6), and oral informed consent was obtained prior to each interview. All informants were informed that their participation was completely voluntary and that they could cease or withdraw from participating in the research at any time without any consequence. All identifying informant information was anonymized to protect informant confidentiality. The confidentiality of informants was further protected by storing all data on an external hard drive that was secured in a locked drawer to which only the researcher, YM, had access.

## 3. Results

A total of 18 *ban zhu ren* in Anhui province and 9 in Zhejiang province were recruited. The informants consisted of 12 females and 15 males. 21 informants worked in public schools and 6 in private schools. The size of the schools varied from 500 students to 3500. The informants’ ages ranged between 20–60 years of age. 7 informants had worked as *ban zhu ren* for more than ten years. All informants were responsible for classes of 30 or more students. The socio-demographic background of informants is detailed in Table 2.

Using an iterative content analysis approach (described in Section 2.4, Data Analysis), the results of the data collection were ultimately grouped according to seven categories/themes: (i) informant perception of the prevalence of mental illness among adolescents; (ii) informant classification of student mental health issues; (iii) informant understanding and labeling of student behavioral issues; (iv) ways that informants determine mental health issues among their students; (v) informant interventions for students they believe to have mental health issues; (vi) informants’ perceived ability to intervene with student mental health issues; and (vii) training informants received on intervening with student mental health issues (Table 1). In grouping results under these seven categories, we assess the raw informant responses, as well as the frequency of agreement of informant perspectives within each category. Any variations between the influence of the demographic variables of age, sex, and years as a *ban zhu ren*, are also detailed within the results of each theme.

### 3.1. Informants’ Perception of the Prevalence of Mental Illness among Adolescents

When asked how common they thought mental health issues were among adolescents, 19 informants stated that mental illness was common among adolescents, and that there had been an increase in the number of students with mental health issues over time. However, all informants agreed that it was necessary to pay attention to student mental health issues in school.

*“Now more and more students have mental health issues. In the past these were unusual. However, in recent years, issues such as depression have increased, and every year there are several students who drop out of school or stay home because of mental illness.”*

(Female informant from Zhejiang province, #10)

*“I think mental health problems are common in adolescents. In my experience, I have encountered students with mental health issues in every class.”*

(Male informant from Anhui province, #27)

Both of these informants voice a common perception among the informants of an increasing prevalence of mental health issues. In general, informants in the 30–39-year-old age group, females, and those with 10 or fewer years of experience as a *ban zhu ren* were more likely to perceive mental illness among adolescents as being common. Given the perceived prevalence of mental illness, it is important to identify exactly what informants consider to be student mental health issues.

### 3.2. What Informants Identify as Student Mental Health Issues 

When asked to list what they consider to be mental health issues, informants report that students who: are “tired of learning”; display “interpersonal problems”; are “addicted” to the internet; are “rebellious”; “fall in love” at a young age; and physically “fight with classmates”, all reflect signs of mental illness, even though in and of themselves, none of these would be considered to be a symptom of mental illness by mental health professionals. Specifically, 14 informants consider that a student who does not want to attend school or does not pay attention in the classroom is “tired of learning”; 12 informants believe that students who do not get along with most of their classmates have “interpersonal problems”; 11 informants state that students who enjoy playing video games online or who stay up late to play games are “addicted to the internet”, which is a mental health issue; 9 informants report that students who argue with their parents or teachers are “rebellious” and have mental health issues; 5 informants believe that adolescents who are in love display signs of mental illness; and 4 believe that students who physically fight with their classmates display mental illness.

*“Psychological problems are common problems for students, especially those who do not like to learn. When they don’t like to learn, they pursue other activities a little more. For example: falling in love at a young age, surfing the internet and fighting, all of these are psychological problems.”*

(Female informant from Anhui province, #4)

This informant’s perception of what many mental health professionals would consider to be normal adolescent behavior, as “psychological problems”, was repeated by other informants. In general, a majority of informants from all age groups, both males and females, and informants of any number of years as a *ban zhu ren*, agreed that “being tired of learning”; “having interpersonal problems”; and being “addicted to the internet” were all mental health issues. However, informants also identified signs of mental illness that may be more commonly agreed upon by mental health professionals.

*“I believe that reasons for one student’s self-harm are a lack of parents’ love and care, imitating others, and because she broke up with her boyfriend.”*

(Male informant from Zhejiang province, #7)

Although few informants addressed causality, several informants agreed with established signs of mental illness. For example, 8 informants identified students with mental health issues who demonstrated suicidal ideation or had attempted suicide; 13 informants stated that students who demonstrate a decreased interest in life or appeared sad for long periods would be considered to be depressed; and 12 informants identify scratching parts of one’s body with a knife or sharp object as “self-harm”, a mental health issue. Among these 12 informants, 11 specifically witnessed girls and 2 witnessed boys who had engaged in self-harm. A few informants did question causality and explained that informants committed self-harm to imitate someone else (*n* = 3), to ease emotions (*n* = 2), for attention (*n* = 2), due to a lack of parental love and care (*n* = 1), and to escape punishment (*n* = 1). 

In general, informants, regardless of age (except 50–60-year-olds), sex, and the number of years as *ban zhu ren*, identified depression, self-harm, and suicidal ideation or attempted suicide as mental health issues.

However, some established signs of mental illness appeared to be normalized by some informants. For example, only 5 informants considered anxiety to be a mental health problem for students; most informants regarded anxiety as a normal response to the stress of studying and the pressure of assessments in schools in China. Overall, few informants identified actual mental health issues in those students who had already been diagnosed by a professional. For example, only 3 informants identified students that were professionally diagnosed with mania, and only a single informant mentioned students professionally diagnosed with ADHD, bipolar disorder, social phobia, or schizophrenia. 

### 3.3. What Do Informants Understand and Label as Behavioral Issues 

Informants were asked what they would classify specifically as a behavioral issue, rather than as a sign of mental illness. Nearly half of informants (*n* = 14) stated that students who do not prepare homework carefully or who do not obey school rules display behavioral problems. Physically fighting with classmates (*n* = 11), interpersonal problems (ie; not getting along with fellow classmates) (*n* = 10), rebelliousness (demonstrated by arguing with parents or teachers) (*n* = 5), inability to control one’s behavior (*n* = 5), using foul language (*n* = 4), bullying (*n* = 2), lying (*n* = 2), stealing (*n* = 2), demonstrating violent tendencies (*n* = 1), demonstrating immature thoughts and behaviors (*n* = 1), and lack of hygiene (*n* = 1), were all identified as reflecting a behavioral, rather than a mental illness, issue. Furthermore, not obeying school rules, physically fighting with classmates, and interpersonal problems were identified as behavioral issues by informants, regardless of their age group, sex, or years as a *ban zhu ren*.

However, 8 informants perceived self-harm as a behavioral problem, and 5 of these also labeled self-harm as a mental health issue. Furthermore, whereas 5 informants believed that falling in love during adolescence was a mental health issue, 4 other informants identified this as a behavioral issue. Self-harm and falling in love during adolescence were also identified as behavioral issues by informants regardless of their age group, sex, or years as a *ban zhu ren*. Additionally, some informants believed that ADHD (*n* = 1), eating paper (*n* = 1), showing no enthusiasm for life (*n* = 1), and having suicidal ideation (*n* = 1) were all behavioral issues.

*“As for behavioral problems I would include: fighting, always getting into arguments with people, not communicating, cutting wrists, and there are lots of others.”*

(Female informant from Zhejiang province, #8)

As with the informant above, several informants conflated what might normatively be agreed upon as behavioral issues with signs of mental illness. The challenges of differentiating between mental health and behavior are further exemplified by the belief of almost all informants (25/27) that behavioral problems are related to mental health, that psychological problems will always lead to behavioral problems (*n* = 9), that mental health problems can be identified by students’ behavior (*n* = 6), and that behavioral problems will lead to mental illness (*n* = 1).

*“I think behavioral problems are related to mental health problems, some behavioral problems are caused by mental health problems, some behavioral problems are the external manifestations of psychological problems.”*

(Male informant from Zhejiang province, #7)

### 3.4. How Informants Identify Mental Health Issues among Their Students

Almost all informants (*n* = 25) reported that they identified student mental health issues by observing students or by speaking with them. Informants also reported that they would identify mental health issues based on parental reports of students’ behavior at home (*n* = 17), feedback from classmates (*n* = 13), feedback from other teachers (*n* = 5), and when students came to them with their problems (*n* = 4). Observing and speaking with students, and feedback from parents and from other students, were used to identify mental health issues by informants, regardless of their age group, sex, or years as a *ban zhu ren*.

*“I think this girl may have mental health issues because of her strange performance. I observed that the girl had pulled out her hair and she had a bald patch. I learned more about her by talking with her mother, for example, she locks herself in her room, so I believe she has mental health issues.”*

(Female informant from Zhejiang province, #8)

However, only 8 informants from 6 of the schools reported they had used any psychological screening tools to identify student mental health. These were predominantly reported by female informants in the 30–39-year-old age group, who served as *ban zhu ren* for 5 years or less. Mental health screening was reported to have been conducted across all 6 schools to help teachers identify mental health problems, according to the requirements of the China Board of Education, although informants could not specify the content or type of the screening tools employed. 

### 3.5. What Informants Have Done about Students They Believe to Have Mental Health Issues 

All informants claimed that they had intervened with what they perceive to be issues of students’ mental health. They report having talked with students and having tried to encourage them to overcome their issues, in addition to trying to put students at ease. One informant reported meeting with a student, whom she believed to have mental health issues, every 2 days to encourage her to overcome her issues (female informant from Zhejiang province #8).

*“At first, I observe the students and if there are problems, I will communicate with the student and others close to the student, like parents and other teachers.”*

(Female informant from Zhejiang province, #11)

Informants report having intervened with students’ parents (*n* = 23), classmates (*n* = 14), their teaching colleagues (*n* = 12), and with school psychological counselors (*n* = 11) to address the mental health issues they believe their students have. In fact, a majority of informants, regardless of their age group, sex, or years as a *ban zhu ren*, report informing parents.

*“When I think that a student has mental health problems, I chat with them to get their trust. I help them to work through their feelings so that their situation improves.”*

(Male informant from Zhejiang province, #7)

Given the amount of time that *ban zhu ren* spend with their students, they are the most likely teacher to win a student’s trust. Informants also discussed how they have intervened with students that they perceive to have mental health issues:

*“I helped a student with mental health problems by working with him to better integrate into the class group, and I keep an eye on this student.”*

(Female informant from Zhejiang province, #10)

Informants report that they have also intervened by educating their students about mental illness (*n* = 20), and by taking students out to the playground, or showing movies in the classroom, especially when students seemed to be stressed (*n* = 17). However, informants report that these activities were not very effective.

*“Occasionally, I will take students to the playground in the evening, and they lie on the ground to see the evening sky. But I don’t know if this helps. I don’t know enough about psychology. So, I tend to talk to the students about my own experience.”*

(Female informant from Anhui province, #2)

Few informants reported intervening by referring students for a professional assessment. Although 12 informants stated that students should be sent to a hospital or clinic if they exhibit “extreme behaviors”, such as self-harm, only 9 informants reported that they had referred students they believed to have mental health issues for professional help.

*“Psychiatric hospitals are not good places, especially for young people, for example, the parents of one of my students brought him to our county mental hospital for examination and his condition became worse. Generally, I would not recommend [that] parents take students to mental hospitals. Whether the child has a problem or not, if the child is taken to the mental hospital I believe it will have a negative psychological impact on the child.”*

(Male informant from Anhui province, #20)

### 3.6. Informants’ Perceived Ability to Intervene with Student Mental Health Issues 

Overall, informants reported having considerable difficulty working with student mental health issues, regardless of the informant’s age group, sex, or years working as a *ban zhu ren*. Specifically, informants identified that they: (i) do not have access to effective ways to intervene (*n* = 7); (ii) lack mental health expertise and the ability to intervene with student issues (*n* = 6); and (iii) have no time or energy to intervene with student mental health issues (*n* = 4).

*“Our curriculum is tight and the school pays more attention to student learning, so we dedicate most of our time to teaching.”*

(Male informant from Zhejiang province, #11)

*“I don’t know how to work with students’ mental health problems and I believe that I need to improve my ability to work with such problems.”*

(Male informant from Zhejiang province, #24)

Furthermore, 7 informants felt that students were generally unwilling to communicate with them about their problems, and 8 reported that parents could not cooperate with them when their child was having problems. 

### 3.7. Informant Training in Student Mental Health 

The final category examines the actual training that informants completed in mental health. Mental health training was not uniform among informants. Although 8 informants reported not having had any mental health training, 19 informants reported they had attended some form of teacher training that included mental health (Table 3). For example, Zhejiang province requires that all teachers attend “C-certificate” training, which consists of a series of lectures concerning mental health, with an examination that is required to be passed for certification. However, in Anhui province, informants reported they received very limited training in mental health. There appears to be no relationship between training and informants’ age or sex, although there appear to be slightly fewer informants who worked 5 years or less as a *ban zhu ren* who received training, compared to those who worked as a *ban zhu ren* for a longer period. Of the 19 informants who reported having mental health training, only 11 received training that was specifically focused on mental health, whereas the remaining 8 received teacher training that included limited mental health instruction. Furthermore, 10 of the 19 informants thought their training was of little or no help, including those who participated in the full mental health training program.

*“I don’t think training helps much, because the content is theoretical and it doesn’t teach us how to work with specific mental health issues, and the training is not all about adolescent mental health. It is useless when we have problems with students.”*

(Male informant from Zhejiang province, #7)

Another informant, who completed the full mental health certificate training in Zhejiang province, stated:

*“The mental health certificate training was 7–8 days. During the training, different people gave lectures related to mental health. I thought some lectures were useful, but many were not.”*

(Female informant from Zhejiang province, #8)

## 4. Discussion

This research identifies that the majority of *ban zhu ren* believe that mental health issues are common among their students. Although one of the responsibilities of *ban zhu ren* is to identify and manage students’ mental health, and all informants report having intervened with student mental health, 8 of the 27 informants claimed to have no mental health training at all, and 10 of the 19 informants who did receive training felt that the training was of little to no value. Hence, 18 of the 27 informants felt they either had inadequate mental health training or no training at all, which may explain why more than half of the informants (*n* = 15) found it difficult to work with student mental health issues. 

Yet, even though the majority of informants believed they were inadequately trained and were challenged by working with student mental health, only one-third of informants (*n* = 9) referred students for professional help, which may, in part, be due to their lack of training, in addition to the strong stigmatization of mental illness in China [45,46]. This stigmatization has led to a lack of open discussion and a lack of social awareness about mental health, and has reinforced social shame toward, and distancing from, those with perceived mental health issues [47]. Our findings of low mental health literacy are corroborated by recent research. For example, Huang et al. identify that the recognition of mental illness among the general public was relatively poor in their sample of 1812 Chinese people, exhibiting a lower recognition of schizophrenia relative to depression; though recognition of all mental illness was found to be significantly lower than that found in Western populations [48]. More problematic, in their study of 1123 non-mental health professionals in Hunan province, Wu et al. found that less than 60% of health professionals could identify schizophrenia, depression, or generalized anxiety disorder correctly. Similar to our *ban zhu ren* informants, “half of the participants rated listening or talking with the patient more highly” than accompanying them for professional help or encouraging the person to visit a psychiatrist or psychologist [49]. 

Prejudice, discrimination, and a general lack of public knowledge concerning mental health have resulted in overwhelmingly negative social attitudes toward mental health in China [50]. As a result, students may be ostracized by other students, who may avoid communicating or engaging with a student with mental health issues. This may explain why several *ban zhu ren* believed that they were protecting the well-being of their students by not referring them for professional help, thereby preventing the student from social stigmatization [50]. However, this does not explain why 14 informants report that they spoke with the classmates of students to assist in helping those believed to have mental health issues, even though such an intervention may, in actuality, be exacerbating the stigma from fellow students toward those identified with mental health issues.

However, another possibility for informants’ insistence on intervening, regardless of a lack of trust in their own capabilities, may be due to their belief in their central role in student mental health, which is reinforced by policies that hold *ban zhu ren* responsible [12]. Furthermore, middle school students spend more time with their teachers, including *ban zhu ren*, than with their own families [51,52]. This familiarity may foster students’ trust and desire to seek advice and help from *ban zhu ren* [53,54]. 

Clearly, there are gaps between the responsibilities of informants for the mental health of their students and their knowledge and capacity to handle these responsibilities, which could have serious consequences, particularly in terms of identifying and seeking treatment for mental health issues. Not only are informants diagnosing far more of their students with mental illness than have been professionally diagnosed, but, more importantly, informants demonstrate a lack of differentiation between what they consider to be a behavioral issue and a mental health issue. This is exemplified where the same issues were identified as both behavioral and mental health issues by nearly half of the informants. For example, several informants believe self-harm is both a mental health and a behavioral issue, while several informants identified normal adolescent behaviors, such as falling in love, as both behavioral and mental health issues, even though they would probably not be classified as either by mental health professionals. Then, there are the symptoms that are normatively considered to be signs of mental health issues, such as self-harm and suicidal ideation, that are being classified by some informants as merely behavioral issues, while common adolescent behavioral issues (e.g.; being tired of learning, physically fighting, being rebellious, interpersonal problems and playing video games) [55,56], are being interpreted as signs of mental illness, despite the fact that these behavioral issues are not listed in diagnostic reference manuals for mental illness, such as the DSM-5. 

This confusion between mental illness and behavioral issues is also reflected by the majority of informants (*n* = 25), who believe that behavioral problems are related to mental illness, because, according to some informants, mental health issues cause students to have many of the behavioral problems discussed. Furthermore, several informants regard students’ behavior alone as the standard by which to identify mental illness. The informant’s quote: “*Psychological problems are common problems for students, especially those who do not like to learn. When they don’t like to learn, they pursue other activities a little more. For example: falling in love at a young age, surfing the internet and fighting, all of these are psychological problems*” (female informant from Anhui province, #4) is illustrative. A student may, of course, be “tired of learning” for reasons that range from students having different learning styles to lack of motivation and interest in the subject matter—all normal responses, particularly among adolescents. What is also interesting about this statement is how one behavior that is perceived to be pathological (“tired of learning”) becomes the precursor for other “psychological problems”, which, again, are actually considered normal adolescent behaviors elsewhere.

These beliefs may be explained, in part, by the lack of psychological training for *ban zhu ren*, as well as by the fact that China’s education system demands a greater emphasis on academic performance and success in assessments and college entrance examinations than on students’ mental health. Furthermore, there is an over-reliance on subjective rather than objective measures to determine student health, as only 8 informants stated that they use any form of psychological screening tools to determine students’ mental health, and the majority of these were *ban zhu ren* who had been practicing for 5 years or less. In addition, the training standards for school psychological counselors are questionable, as these are often schoolteachers who have received no additional psychological training [14]. 

Another possible explanation for this lack of differentiation between non-conformist behavior and mental illness may also be a cultural perception of what is normal behavior and what is mental illness, which is relative to a given culture. For example, Huang et al. identify that “culturally specific idioms of mental illness provide a socially accepted interpretation of symptoms (i.e., ‘excessive thinking’)” [48]. Hence, it is also worthwhile to consider the relationship between some of the behaviors identified by informants as mental illness and what may, in actuality, be issues of social conformity in a given context. For example, we may ask why mental illnesses are being considered by informants when adolescents are “tired of learning”, “playing video games”, or “falling in love at a young age”? Might these fit more with issues of social non-conformity that are, in effect, being medicalized, as Szasz (1960, 1997) argued about the medicalization of nonconformist behavior by the state [57,58]? The medicalization of adolescence has been identified across issues ranging from the diagnosis of ADHD [59,60,61], through deviance in the juvenile justice system [62], to what are primarily social issues [63]. Thus, labeling the nonconformist behaviors of adolescents as pathological can also be understood as a process of medicalization [64,65,66,67,68]. Hence, in addition to the lack of informants’ capacity to work with the full spectrum of adolescent needs in their schools, we could also question whether the informants’ lack of differentiation between what may be perceived as the normal behaviors of adolescents and mental illness is a result of medicalizing student non-conformity. These are important questions to consider, if student mental health in China, and in other contexts where those responsible for adolescent mental health are not appropriately trained, is to be fully and competently addressed.

On the other hand, it should be acknowledged that some informants did identify symptoms of mental health issues (e.g., appearing to be depressed (*n* = 13), evidence of self-harm (*n* = 12), and suicidal ideation (*n* = 6)) that are corroborated by psychological reference manuals, such as the DSM-5. Similarly, some informants identified behaviors that are normatively considered to be behavioral issues, such as disobeying school rules (*n* = 14), physically fighting with classmates (*n* = 11), and interpersonal problems (*n* = 10) [55,56].

Thus, the issue is not whether *ban zhu ren* or any middle-school staff member in any context should be responsible for the mental health of their students, but rather whether they have been given the skill sets and tools whereby they can effectively intervene and know when to refer students to mental health professionals. However, the informants of this study are by no means outliers. The management of student mental health is a challenge for teachers in many countries. Studies in the UK and Germany emphasize how teachers feel unqualified to support students with mental health issues [69,70,71]. For example, a study of 291 primary schools in the UK identified that although teachers expressed great interest in children’s mental health, they received little training and expressed uncertainty about pathways of referral [72]. 

The literature has frequently championed the impact of proper mental health training [73]. In a study concerning in-depth mental health first-aid training in Australia, teachers’ knowledge was increased, and their understandings of treatment were more akin to those of mental health professionals [74]. Furthermore, training was found to increase confidence in providing help to students and colleagues [74]. However, the majority of informants in our study reported difficulties in intervening with student mental health, even after training. Hence, the length, depth, and comprehensiveness of training may impact teacher effectiveness [75]. Furthermore, there may be a separate issue of confidence and professional identity. In a study in Chongqing, China, appropriate occupational training markedly improved the teachers’ sense of their role, professional behavioral tendency, occupational values, and sense of belonging [76].

However, this is not to imply that training and responsibilities should be an individual matter. Several studies emphasize the importance of collaborative teamwork among colleagues, both in schools and across the community, to address student mental health. A Norwegian study identified the positive outcomes when teachers and mental health professionals work together to support student mental health [77]. Similarly, a Canadian study demonstrated that cooperation among teachers, social workers, and psychologists was effective in supporting student mental health [78].

Yet, even if all of these factors were rectified, challenges may persist due to the social stigma of mental illness in China, as well as in myriad contexts around the world. Redressing social stigma is a long process, although a global review of the literature identifies that social contact is the most effective type of intervention to improve stigma-related knowledge and attitudes, at least in the short term [79].

## 5. Conclusions

In summary, this research was a preliminary attempt to investigate the perceptions and actions of a group of *ban zhu ren* concerning the mental health of their students. This study identifies that our sample of *ban zhu ren* commonly rely on their own perceptions, which are often independent of what is accepted and practiced by mental health professionals, for their determinations of student mental health issues. This is one of the first studies concerning the perceptions and actions of *ban zhu ren* regarding student mental health in China, though there are a number of limitations to this preliminary study. Given the small sample of informants from a small number of schools, the findings are not and cannot be generalized to different educational systems, educational settings, or other cultural frameworks, nor even to different schools within the two provinces studied, much less throughout China. Furthermore, the size of the sample does not engender meaningful analysis of the impact of various demographic factors on responses, such as age, sex, and years working as a *ban zhu ren*, which all yielded similar results in this sample. The methodology for the study was also a limitation, particularly in terms of protecting against bias. For example, the convenience sample did not strive for representativeness. The data collection instrument was piloted, but not validated, and, although content analysis is a structured process, it is very much a subjective process in which “excessive interpretation” can threaten validity [43]. Lastly, informant veracity may have been compromised, due to the sensitivity of the study questions. 

Given the importance of adolescent mental health and the dearth of research on the influence of those who are most likely to intervene with adolescents, more research in this area is necessary to better understand the issues identified in this research. Future studies in this important area will benefit from mixed-methods research of higher internal and external validity and significantly larger and representative samples. Prioritizing the mental health training of *ban zhu ren* in China will not only benefit student mental health, but may also help to reduce the long-standing social stigma of mental illness in China.

## Figures and Tables

**Table 1:** Generation of categories and sub-categories from initial coding using content analysis.

Organizing Phase			Preparatory Phase	
Categories		Sub-Categories		
**Step 5:** **Group Codes into Clusters/** **Content Areas**	**Step 4:** **Identify Categories or Themes**	**Step 3:** **Perform Initial Coding**	**Step 2:** **Identify Key Words, Thoughts, and Concepts**	**Step 1:** **Initiate Textual Analysis with Multiple Readings of the Text**
Informants’ perception of student mental health issues	Informants’ perception of the prevalence of mental illness among adolescents	How common do informants believe mental health issues were among adolescents	Adolescent mental health issues are worsening	
		Perception of need to pay attention to student mental health	It is necessary to pay attention to student mental health	
	What informants identify as student mental health issues	Mental health issues classified by informants	Depression, self-harm	
Informants’ differentiation of mental health and behavioral issues among adolescents	What do informants understand and label as behavioral issues	Informants’ classification of behavioral issues	Physical fighting, interpersonal problems	
		Informants’ understanding of behavioral issues and mental health issues	Behavioral problems are related to mental health	
		Informants’ rationale of a relationship between behavioral and mental health issues	Psychological problems will always lead to behavioral problems	
Informants’ intervention in student mental health issues	How do informants identify mental health issues among their students	How informants gather information to identify students’ mental health	Feedback from parents	
		Criteria informants use to identify mental health issues	Identify mental health issues based on student behaviors	
	What informants have done about students they believe to have mental health issues	Interventions carried out by informants	Help students with mental health issues to establish a closer relationship with peers.	
		Informants’ decision to refer students to a mental health professional	I have deliberately not referred to a psychological clinic	
Informants’ capacity for student mental health intervention	Informants perceived ability to intervene with student mental health issues	Informants perceived ease or difficulty in intervening with student mental health	It is difficult to work with student mental health issues	
		Informants’ perceived barriers of intervening with student mental health	No time to work with student mental health issues	
	Informant training in student mental health	Specific type and extent of informant mental health training	“C-certificate” training, with a series of lectures concerning mental health, that concludes with an examination	
		Informants’ perceived impact of the training	Training does not help	

**Table 2:** Socio-demographic background of the sample.

Socio-Demographic Factors	*n* (%)
Location of school	
Anhui Province	18 (66.7)
Zhejiang Province	9 (33.3)
Sex	
Female	12 (44.4)
Male	15 (55.5)
Age	
20–29	7 (25.9)
30–39	10 (37.0)
40–49	7 (25.9)
50–60	3 (11.1)
Type of school where informants worked	
Public	21 (77.8)
Private	6 (22.2)
Grade taught	
Seventh grade	9 (33.3)
Eighth grade	10 (37.0)
Ninth grade	8 (29.6)
Years teaching	
1–5	7 (25.9)
6–10	7 (25.9)
11–20	5 (18.5)
>20	8 (29.6)
Years as a *ban zhu ren*	
1–5	12 (44.4)
6–10	8 (29.6)
11–20	4 (14.8)
>21	3 (11.1)
The number of students in class	
30–39	11 (40.7)
40–49	10 (37.0)
50–59	6 (22.2)
Subject taught	
Mathematics	12 (44.4)
English	4 (14.8)
History	4 (14.8)
Politics	3 (11.1)
Chinese	2 (7.4)
Science	2 (7.4)

**Table 3:** Reported informant training and perceived usefulness of training in mental health.

Reported Training	Number of Informants
Training specifically for mental health	11
Training was of little or no help	10
Training included some mental health training	8
No training	8

## Data Availability

The datasets supporting the results of this research are available from The Center for Global Health at Zhejiang University.

## Supplementary Materials

The following are available online at https://www.mdpi.com/article/10.3390/ijerph18147271/s1, File S1: The Socio-demographic Information of Informants, Table S1: The Interview Schedule.
