# Supplementary material for: Teachers’ Perceptions of Student Mental Health in Eastern China: A Qualitative Study
Source: Int J Environ Res Public Health. 2021 Jul 7;18(14):7271. doi: 10.3390/ijerph18147271 (PMC8303108; doi:10.3390/ijerph18147271)
Supplement: Supplementary file 1 [file ijerph-18-07271-s001.zip › ijerph-1221796-supplementary.pdf]

Supplementary Materials

File S1: The Socio-demographic Information of Informants

Sex: ________ Age: _______ School: _________________________ Grade taught: ___________

Subject: ____________ Length of time as a teacher: _________ Length of time as *ban zhu ren*: ___________

Number of class students: _____________ University Degree: _____________

Place of birth[urban/rural]: ____

Place of residence[urban/rural]: ____

Marital status: unmarried/married/divorced/remarried

Number of the class teacher's children: ___________ Age of their children: ______________

Table S1. The Interview Schedule.

QUESTIONS ALIGNED WITH RESEARCH QUESTIONS	RQ1: Do middle school teachers identify mental health issues in their students, and if so how? What criteria do they use for this label?	RQ2: How do middle school teachers differentiate behavioral issues from mental health issues?	RQ3: How do middle school teachers support students who have mental health issues?	RQ4: What knowledge do middle school teachers believe they have that helps them to support students whom they identify as having mental health issues?
What do you consider to be mental health issues?	✔			
In your experience, do you think mental health issues are common among adolescents? If so, please explain why?				
Do you think it is necessary to pay attention to the mental health of students? Who do you think should do this work in a school, and why?	✔			
What are some examples you have of student mental health issues, and which is the most commonly encountered in the classroom?	✔			
How do you determine when a student has a mental health-related issue?	✔			
Why do you think these are mental health issues?	✔			

What do you consider to be student behavioral issues?		✔		
What are some examples you have seen of student behavioral issues, and which of these are the most common?		✔		
Do you think behavioral issues are related to mental health issues, and why?		✔		
How can you tell the difference between what you are calling a mental health issue and a behavioral issue?		✔		
What will you do when you find a student has what you believe are mental health issues?			✔	
What have been the outcomes of these activities and do you think these have been helpful, why or why not?			✔	
Is there anyone at your school or in the community from whom you can get advice or information about mental health problems? If so, to whom do you turn and why?			✔	
Have you experienced barriers to help students whom you believe to have mental health issues?			✔	
Why do you believe you experience these barriers and what is needed to help overcome these barriers?			✔	
At what point will you think that a student			✔	

needs professional help, and a referral for mental health services?				
How do you decide to whom you would refer a student that you believe has mental health issues? To whom have you referred your students in the past and why?			✔	
Have you shared knowledge about mental health with students? Have you ever intervened in the mental health issue of students?			✔	
Do you think you have the capability to manage student mental health issues and why?				✔
Please tell me if you have received any special training that would help you work with students whom you believe to have mental health issues? How has this training helped you handle mental health issues in the classroom（frequency/effects/official arrangements/autonomy）?				✔
If you have not had any training, would you be willing to attend training and why?				✔
Do you think you need extra training to improve your capabilities in managing students with mental health issues?				✔
What kind of training				✔

do you think would be most beneficial in helping you to manage students that you believe have mental health issues and why?	
Can you please share any cases of students you worked with that you believe had mental health issues and how you managed these cases, as well as the outcome of your interventions?	
